# Interleukin-1 Receptor-Associated Kinase-2 (IRAK2) Is a Critical Mediator of Endoplasmic Reticulum (ER) Stress Signaling

**DOI:** 10.1371/journal.pone.0064256

**Published:** 2013-05-28

**Authors:** Samir Benosman, Palaniyandi Ravanan, Ricardo G. Correa, Ying-Chen Hou, Minjia Yu, Muhammet Fatih Gulen, Xiaoxia Li, James Thomas, Michael Cuddy, Yasuko Matsuzawa, Renata Sano, Paul Diaz, Shu-ichi Matsuzawa, John C. Reed

**Affiliations:** 1 Sanford-Burnham Medical Research Institute, La Jolla, California, United States of America; 2 The Cleveland Clinic Foundation, Department of Immunology, Lerner Research Institute (NB30), Cleveland, Ohio, United States of America; 3 University of Texas, Southwestern Medical Center, Dallas, Texas, United States of America; Wayne State University, United States of America

## Abstract

Endoplasmic reticulum (ER) stress occurs when unfolded proteins accumulate in the lumen of the organelle, triggering signal transduction events that contribute either to cellular adaptation and recovery or alternatively to cellular dysfunction and death. ER stress has been implicated in numerous diseases. To identify novel modulators of ER stress, we undertook a siRNA library screen of the kinome, revealing Interleukin-1 Receptor-Associated Kinase-2 (IRAK2) as a contributor to unfolded protein response (UPR) signaling and ER stress-induced cell death. Knocking down expression of IRAK2 (but not IRAK1) in cultured mammalian cells suppresses ER stress-induced expression of the pro-apoptotic transcription factor CHOP and activation of stress kinases. Similarly, RNAi-mediated silencing of the IRAK family member Tube (but not Pelle) suppresses activation of stress kinase signaling induced by ER stress in *Drosophila* cells. The action of IRAK2 maps to the IRE1 pathway, rather than the PERK or ATF6 components of the UPR. Interestingly, ER stress also induces IRAK2 gene expression in an IRE1/XBP1-dependent manner, suggesting a mutually supporting amplification loop involving IRAK2 and IRE1. *In vivo*, ER stress induces *Irak2* expression in mice. Moreover, *Irak2* gene knockout mice display defects in ER stress-induced CHOP expression and IRE1 pathway signaling. These findings demonstrate an unexpected linkage of the innate immunity machinery to UPR signaling, revealing IRAK2 as a novel amplifier of the IRE1 pathway.

## Introduction

The human genome encodes 727 kinases and kinase-like proteins, of which 528 are known or suspected protein kinases [1]. It has been estimated that only approximately 10% of kinases have been studied in detail, despite the clear promise of this class of drug targets for therapeutics development. At present, 185 of the human kinases are the subject of ≤10 publications and 58 kinases have no associated publications [2]. Thus, a need exists to define the full repertoire of biological contexts in which kinases function in health and disease.

The cellular response to environmental stress represents a situation where kinase-mediated signaling transduction plays critical roles in cellular adaptation. For example, accumulation of unfolded or misfolded proteins in the Endoplasmic Reticulum (ER) triggers various signal transduction cascades, collectively known as the unfolded protein response (UPR). ER stress occurs in many disease contexts, and is increasing recognized as a contributor to the pathology of cancer, neurodegenerative diseases, diabetes, heart disease, and inflammation [3]. In unstressed cells, ER-associated chaperones facilitate the folding and maturation of proteins imported into the ER. These chaperones also act as sensors of defects in protein folding. For example, Grp78 (BiP), a member of the Hsc70/Hsp70 family, interacts with three ER-associated signaling molecules: IRE1, PERK and ATF6 [4]. Upon accumulation of misfolded proteins, Grp78 releases these initiators to trigger the UPR.

The ER membrane-associated protein IRE1 (which possesses both kinase and endoribonuclease activities) induces unconventional splicing of the mRNA encoding the transcription factor XBP1, leading to production of XBP1 protein. XBP1 is a transcription factor that induces expression of UPR genes that include chaperones and protein degradation factors. PERK phosphorylates and inhibits the translation initiating factor eIF2α, thereby stopping further protein translation to reduce strain on the ER. Membrane-tethered ATF6 translocates to the Golgi where it is cleaved by proteases to liberate the active ATF6 transcription factor, which induces expression of UPR genes. If the UPR fails to restore ER homeostasis, other pathways activated by IRE1, PERK, and ATF6 begin to move the cell towards death. For example, XBP1, PERK (via ATF4) and ATF6 induce expression of CHOP, a transcription factor that modulates expression of genes encoding Bcl-2 family proteins and TRAIL Receptors [5]. IRE1 also activates Apoptotic Signaling Kinase-1 (ASK1) via the adaptor TRAF2. ASK1 induces the activation of stress kinases JNK and p38MAPK, which contribute to cell death by phosphorylating Bcl-2 family proteins and other mechanisms [6].

The Interleukin-1 Receptor Associated Kinase (IRAK) family comprises 4 members in humans: IRAK1, IRAK2, IRAK3(IRAK-M) and IRAK4. These proteins have been described as modulators of NF-κB signaling via Toll-like receptors (TLRs) [7]. Activated TLRs recruit IRAK4 via the adaptor MyD88. IRAK4 then phosphorylates IRAK1, which recruits TRAF6 and activates NF-κB and JNK through TAK1 phosphorylation [8]. IRAK2 and IRAK3 have been shown to modulate TLR signaling [9], [10], however, their mechanisms of action remain elusive as these proteins apparently lack intrinsic kinase activity.

In our effort to identify novel modulators of ER stress-induced cell death, we undertook a kinome siRNA library screen for targets that are required for the cytotoxic activity of an ER stress-inducing compound in human cancer cell lines. Among 707 kinases screened, we identified IRAK2 as a new contributor to ER stress-induced pathways that promote cell death. No previous study has reported the involvement of IRAK family kinases in ER stress. In the present study, we documented a novel role for IRAK2 in ER stress responses, linking IRAK2 to the IRE1 pathway.

## Results

### IRAK2 Identified in siRNA kinome Library Screen for Suppressors of ER-stress Induced Cell Death

**Figure 1:**
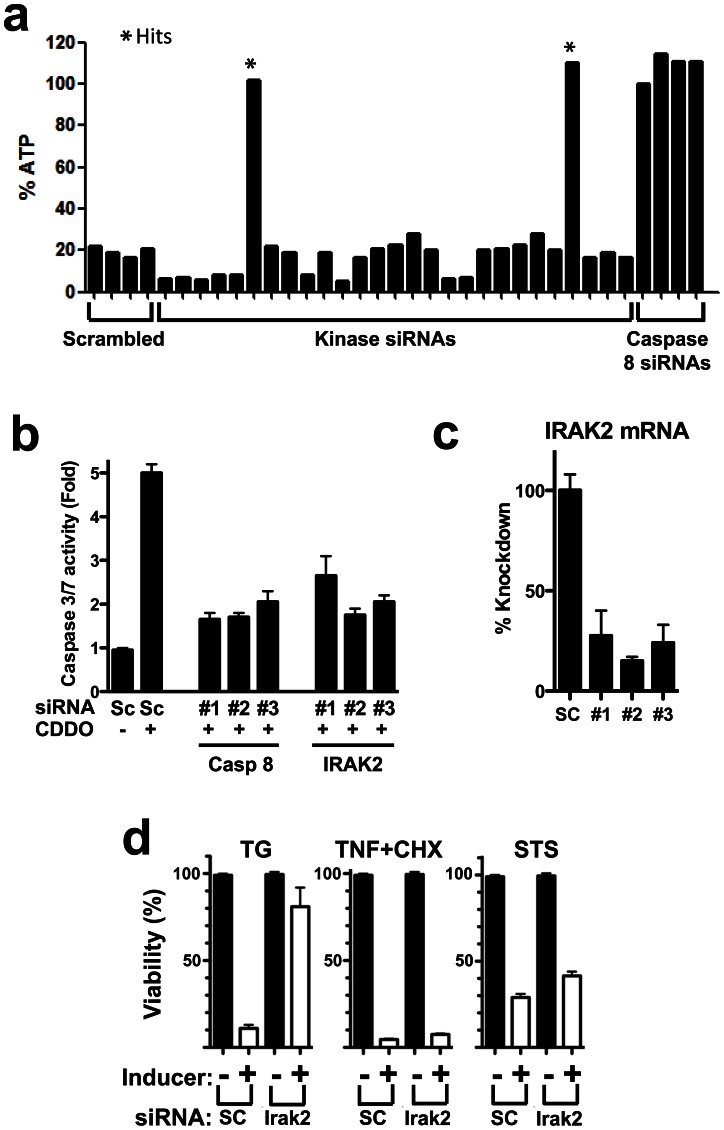
IRAK2 knock down selectively inhibits ER stress-induced cell death. (**a**) Hits identified by siRNA kinome library screening after treatment of PPC1 cells with CDDO-Im for 24 hrs. Representative data are shown from an assay plate. Cell viability was estimated by measuring cellular ATP levels (% relative to untreated control). Asterisks represent the hits obtained with IRAK2 siRNAs. Caspase 8 siRNAs were employed as positive controls and scrambled RNAs as negative controls. (**b**) PPC1 were transfected with scrambled (SC) or 3 independent siRNAs targeting IRAK2 or Caspase 8 (positive control) then treated with CDDO-Im (1 µM) for 3 hrs. Cells were then lysed and Caspase 3/7 activity as measured. Data are expressed as fold induction from the control. Data are mean ± std dev (SD), n = 3. (**c**) IRAK2 siRNA Knockdown was verified by measuring IRAK2 mRNA expression by qRT-PCR. Data are mean ± SD, n = 3. (**d**) IRAK2 is selectively involved in ER stress-induced cell death. PPC1 cells were transfected two siRNAs targeting IRAK2 or with scrambled (SC) control siRNA. After 1 day, cells were cultured without (black bars) or with Thapsigargin (TG), TNF-α+cycloheximide, or Staurosporine (STS) (white bars) for 24 hrs. Cell viability was estimated by measuring ATP levels, expressing data as % of control untreated cells (mean±SD, n = 3).

We screened a siRNA library covering 707 kinases to identify members of the kinome that contribute to ER stress-induced cell death. The model system employed entailed treatment of human prostate cancer cell line PPC1 with an imidazole derivative of 2-cyano-3,12 dioxooleana-1,9 dien-28-oyl imidazoline (CDDO), a synthetic triterpenoid currently in clinical development [11]. CDDO and structurally related electrophilic compounds stimulate cancer cell apoptosis via mechanisms involving the UPR machinery [12], [13]. Previous studies demonstrated that triterpenoid-induced apoptosis in PPC1 cells is dependent on UPR transcription factor CHOP, Death Receptor 5 (DR5) (a known CHOP target gene), and Caspase-8 (a downstream mediator of DR5-induced apoptosis) [14], prompting us to use Caspase-8 siRNA as a positive control for the screen (Fig. 1a; Fig. S1a and b in File S1). Since kinases were screened at 4-fold coverage (using 4 different siRNAs targeting each kinase mRNA), we ranked the hits based on the number of siRNAs that restored cell survival in CDDO-Im-treated cultures by 50% (Primary hits). Of the 707 kinase targets screened (∼2900 siRNAs tested), 25 produced hits with two or more of the four siRNAs. Upon repeat testing, this number dropped to 7 candidate kinases where at least two of the four siRNAs scored as hits. With qRT-PCR verification of target knockdown by siRNAs that rescued against CDDO-Im (but not siRNAs that failed to rescue), the number reduced to 6 candidate kinases. Further studies showed that siRNA-mediated knockdown of all 6 of these kinases afforded protection of PPC1 cells against another ER stress inducer (Thapsigargin, an irreversible inhibitor of the ER Ca^2+^ATPase, SERCA) but not Staurosporine (activator of the mitochondria pathway for apoptosis [intrinsic pathway]) or TNF-α (activator of death receptor pathway for apoptosis [extrinsic pathway]), thus showing pathway selectivity (Fig. 1d). Of these, only 2 inhibited CHOP activity (Fig. S1a in File S1). Finally, only one of these candidates corresponding to IRAK2 showed cytoprotective activity in several additional tumor cell lines (ALVA31, A549, U251, HeLa) treated with CDDO-Im.

Of the 4 IRAK2-targeting siRNAs tested, 3 rescued against CDDO-Im-induced cell death in multiple tumor cell lines. To clarify whether IRAK2 knockdown affected apoptosis, effector Caspase activity (Caspases-3/7) was measured in cells where IRAK2 was knocked-down using 3 independent siRNAs. All three siRNAs that effectively reduced IRAK2 mRNA levels also reduced CDDO-induced effector Caspase activity to levels comparable to cells transfected with Caspase-8-targeting positive control siRNAs (Fig. 1b and c).

### IRAK2 is Required for UPR Signaling in Mammalian and Insect Cells

**Figure 2:**
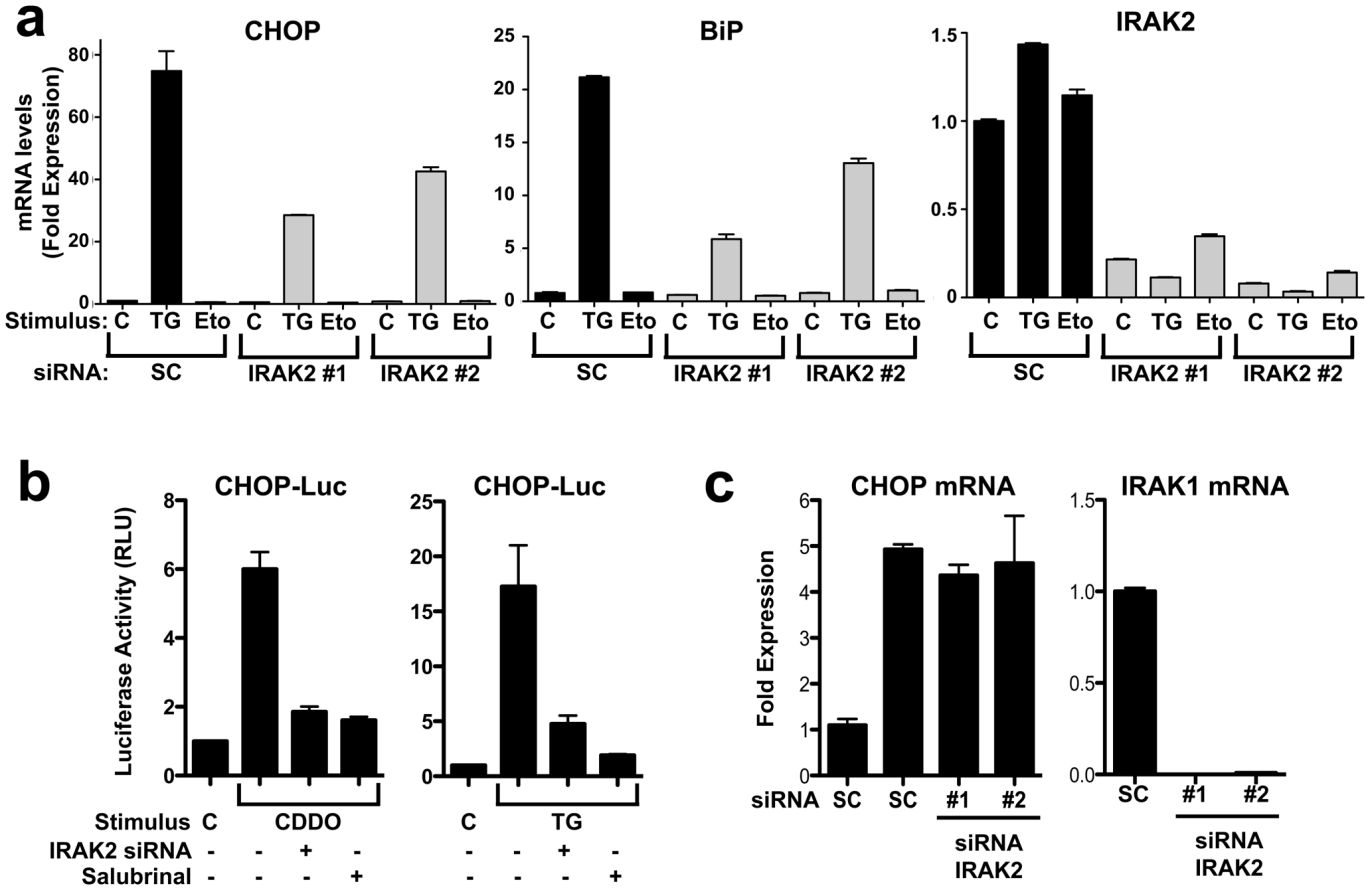
IRAK2 knock down inhibits UPR signaling. (**a**) PPC1 cells were transiently transfected with 2 independent IRAK2 siRNAs (gray bars) or scrambled controls (SC) (black bars). After 2 days, cells were cultured with 5 µM Thapsigargin (TG) or 100 µM Etoposide (Eto) for 3 hrs then total RNA was extracted. Relative levels of mRNAs encoding CHOP (left), BiP/Grp78 (middle), and IRAK2 (right) were compared by qRT-PCR and displayed as ratios relative to a housekeeping gene (cyclophilin). Data are mean±SD, n = 3. (**b**) PPC1 cells were transfected with IRAK2 siRNA (+) or scrambled (Sc) siRNA (−). After 2 days, reporter gene plasmids CHOP-Luc and Renilla-Luc were transfected. Then 4–6 hrs later, cells were cultured without (C) or with 5 µM CDDO-Im (CDDO) or TG, with or without the ER stress inhibitor Salubrinal for 6 hrs. Luciferase activity was subsequently measured, normalizing firefly Luc driven from the *CHOP* gene promoter relative to Renilla Luc and expressing data as fold-induction relative to untreated cells transfected with SC control siRNA (mean±SD, n = 3). (**c**) Cells were transfected with either scrambled or IRAK1 siRNAs and subsequently cultured without (−) or with (+) 5 µM TG for 3 hrs. Then, RNA was extracted and CHOP and IRAK1 mRNA expression were measured by qRT-PCR (normalized to Cyclophilin). Data are expressed as fold-induction relative to untreated SC control transfected cells (mean±SD, n = 3).

Next, we compared UPR signaling events in PPC1 (Fig. 2a) and HeLa (Fig. S2a in File S1) cells in which IRAK2 expression was knocked down by transfection using two different siRNAs. ER stress was induced by Thapsigargin (TG). Compared to cells transfected with scrambled RNA controls, IRAK2 knockdown cells displayed impaired induction of CHOP and BiP(Grp78) mRNAs, as measured by qRT-PCR. Similarly, CHOP promoter activity as measured by luciferase reporter gene assays was dramatically blunted by IRAK2 knockdown in cells treated with ER stress inducers TG or CDDO-Im (Fig. 2b). In contrast, siRNA-mediated knockdown of IRAK1 had no effect on CHOP expression (Fig. 2c). Controls for these experiments included culturing cells with DNA-damaging agent etoposide (which had no effect on CHOP and BiP expression) and with Salubrinal (a compound that suppresses UPR signaling) [15]. Successful knock-down of IRAK2 mRNA was confirmed by qRT-PCR (Fig. 2a,*right panel*).

**Figure 3:**
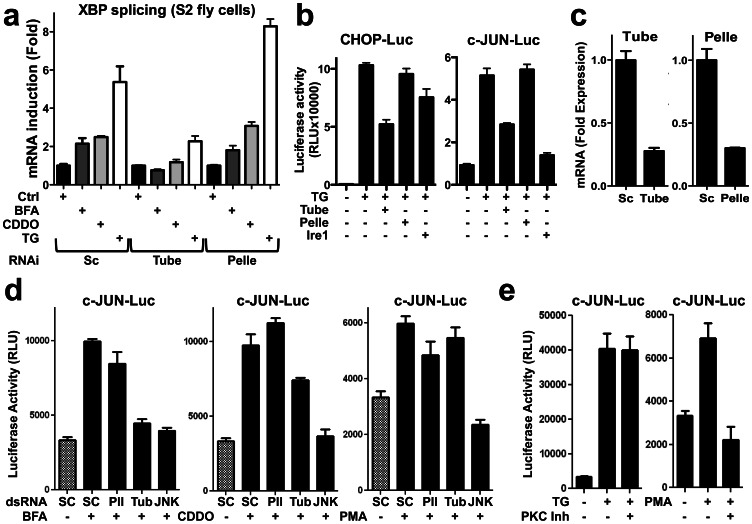
IRAK2 homolog knock down inhibits UPR signaling in fly cells. (**a**) Drosophila S2 cells were transfected with either scrambled, Tube or Pelle dsRNAs and subsequently treated with 2 µM TG, 2 µg/mL BFA or 1 µM CDDO-Im for 3 hrs, then RNA was extracted and relative levels of spliced and unspliced XBP mRNA levels were measured by qRT-PCR (normalized to rp49), reporting the ratio of unspliced XBP1 to total XBP1 mRNA (mean±SD, n = 3). (**b**) S2 cells containing pFR-luciferase reporter gene plasmid were transfected with plasmids encoding chimeric c-JUN (pFA-c-JUN) or CHOP (pFA-CHOP) transcription factors and dsRNA targeting Tube (tub), Pelle (pll), IRE1 or non-specific dsRNAs. After 72 hrs, cells were treated with 100 nM TG or DMSO for an additional 24 hrs. Relative luminescence units (RFU) were measured and normalized for cell viability (mean ± SD; n = 3). (**c**) S2 cells were transfected with either non-specific, Tube or Pelle dsRNAs for 72 hrs. Then, RNA was extracted and relative Tube or Pelle mRNA levels were measured by qRT-PCR (normalized to rp49, mean±SD, n = 3). (**d**) pFR-luciferase stable Drosophila S2 cells were transfected with pFA-c-JUN and dsRNA targeting Tube (tub), Pelle (pll), JNK or non specific DsRNAs (SC), incubated for 72 hours then treated with 0.5 µg/mL Brefeldin A (BFA), 50 nM CDDO-ME, or 2 µg/mL phorbol-12-myristate-13-acetate (PMA) for an additional 24 hrs. Luminescence was measured and normalized by cell viability. Data are mean±SD, n = 3. (**e**) pFR-luciferase stable Drosophila S2 cells were transfected with pFA-c-JUN, incubated for 72 hrs, then treated with 100 nM TG or 2 µg/mL PMA and 5 µM of the PKC inhibitor Go 6983 (PKC inh) for an additional 24 hrs. Luminescence was measured and normalized for cell viability. Data are mean±SD, n = 3.

To determine whether a role for IRAK family genes is evolutionarily conserved, we investigated the two known IRAK homologs of *Drosophila*, Pelle and Tube. For these experiments, fly S2 cells were transfected with double-strand RNAs (dsRNA) corresponding to Pelle, Tube, or IRE1. Silencing Tube (but not Pelle) reduced splicing of endogenous XBP1 mRNA after treatment with various ER stress inducers, including TG, Brefeldin A (BFA) and CDDO-Im (Fig. 3a). Activity of UPR pathways was also measured by reporter gene assays, using chimeric transcription factors that are stimulated upon phosphorylation by c-JUN N-terminal Kinases (JNKs) (measured with c-JUN-Luc) or p38MAPK (measured with CHOP-Luc)(Fig. 3b, c). IRE1 knockdown served as a positive control in the experiments with S2 cells, reducing activity of stress kinases as measured by this reporter gene method –a result that is consistent with the essential role of IRE1 for stress kinase activation during ER stress [16]. Knocking down Tube (but not Pelle) significantly reduced ER stress signaling induced by TG, BFA, and CDDO-Im in fly S2 cells (Fig. 3d). Analysis by qRT-PCR confirmed successful knockdown of mRNAs for the Tube and Pelle targets (Fig. 3c).

In contrast, Tube knockdown had no effect on c-JUN reporter gene activity induced by protein kinase C (PKC) activator, PMA, whereas knocking down JNK (positive control) did suppress c-JUN activity induced by PMA (Fig. 3d). Importantly, treating S2 fly cells with a PKC inhibitory compound (Go 6983) reduced c-JUN reporter gene activity induced by PMA (PKC activator) but did not inhibit TG-induced c-JUN activity (Fig. 3e). These data confirm that the stress kinase response induced by TG in S2 fly cells is due to ER stress and not ER Ca^2+^ mediated PKC activation. Thus, as in mammalian cells, certain IRAK family members contribute to UPR signaling in fly cells while others do not (e.g. Tube but not Pelle).

### IRAK2 is Required for IRE1 Pathway Signaling using ER Stress Responses

**Figure 4:**
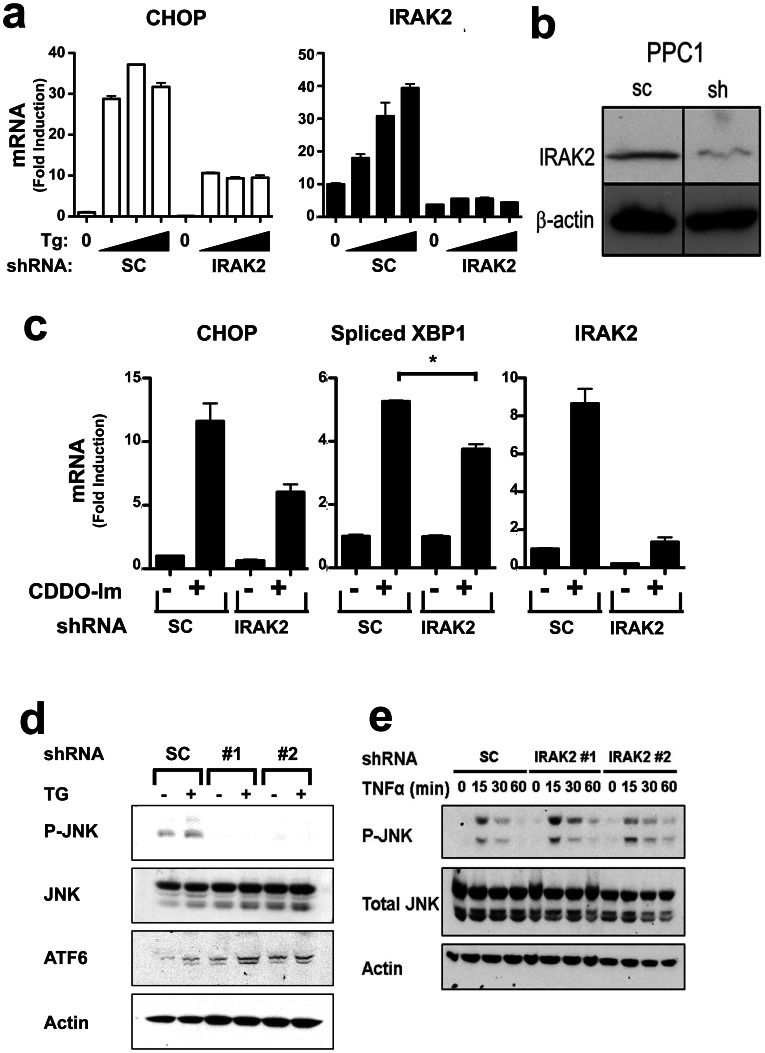
IRAK2 knock down by shRNA vectors reduces ER stress signaling. (**a**) PPC1 cells were stably infected with recombinant lentiviruses producing IRAK2 or scrambled (SC) shRNAs. After selection of stable clones, cells were cultured with various concentrations TG (1.6, 5, or 15 µM) for 3 hrs, then total RNA was extracted. Relative levels of CHOP (left) and IRAK2 (right) mRNAs were measured by qRT-PCR. Data represent fold induction relative to untreated cells, normalized relative to housekeeping gene, cyclophilin (mean±SD, n = 3). (**b**) IRAK2 protein levels were compared in PPC1 cells stably infected with empty vector versus IRAK2 shRNA lentiviruses. Cell lysates were normalized for total protein content and analyzed by SDS-PAGE/immunoblotting using antibodies specific for IRAK2 (top) or β-actin (bottom). (**c**) PPC1 cells stably expressing IRAK2 or Sc shRNAs (see above) were cultured with (+) 5 µM CDDO-Im or with DMSO (-) for 3 hrs, then relative levels of CHOP (left), Spliced XBP1 (middle), or IRAK2 (right) mRNAs were measured as described above (mean±SD; n = 3). Statistical significance of spliced XPB-1 mRNA data was confirmed by two-tailed unpaired *t* test (*, p = 0.0125). (**d,e**) PPC1 cells stably expressing scrambled or IRAK2 shRNAs were cultured with 5 µM Thapsigargin (TG) for 3 hrs (d) or 100 ng/mL TNF-α for 0–60 min (e) Lysates were prepared, normalized for total protein content, and analyzed by immunoblotting using antibodies specific for phosphor-JNK1, total JNK1, cleaved ATF6, or actin. Data representative of 3 or more experiments.

To verify these results by another method, we generated cancer cell lines in which IRAK2 expression was stably knocked-down in PPC1 cells using recombinant IRAK2-targeting shRNA lentiviruses and compared them to cells harboring scrambled shRNA control vectors. Molecular analysis confirmed robust suppression of IRAK2 mRNA expression (Fig. 4a) and IRAK2 protein (Fig. 4b) in cells infected with IRAK2 shRNA viruses. In addition, the selectivity of the IRAK2 knockdown by shRNA was confirmed by q-RT-PCR comparison with other IRAK family members (Fig. S2b in File S1).

Similar to transfection of siRNAs, CHOP gene expression induced by ER stress agents TG or CDDO-Im was markedly reduced in IRAK2 shRNA knock-down cells (Fig. 4a, c). ER stress-induced splicing of XBP1 mRNA was also blunted in PPC1 cells with stable shRNA-mediated knockdown of IRAK2 expression (Fig. 4c), suggesting an effect on IRE1 signaling. Consistent with an impact on IRE1 signaling, activation of stress kinases by ER stress inducer TG was also suppressed in IRAK2 shRNA knock-down cells (Fig. 4d), but not in cells stimulated with cytokine TNF-α (Fig. 4e) and not by phorbol-12-myristate-13-acetate (PMA) (not shown), as measured by phospho-antibody immunoblot analysis of JNK. Lower levels of p-JNK seen in IRAK2 deficient cells prior to stimulation with TG may reflect the background levels of ER stress that occur in the hyper-oxygenated environment of routine tissue where atmospheric O_2_ pressures exceed in vivo tissues levels. In contrast to CHOP and stress kinases, IRAK2 knockdown did not impact ATF6 proteolytic processing (Fig. 4d). Importantly, stable IRAK2 knockdown also did not alter the expression of UPR-independent genes such as NQO1 (oxidative stress-induced), c-JUN (Ca^2+^-induced), and ATG12 (autophagy-induced) (Fig. S3a in File S1). Thus, IRAK2 appears to selectively modulate the IRE1 pathway among UPR signaling events.

### *IRAK2* Gene Expression is Induced by ER Stress

**Figure 5:**
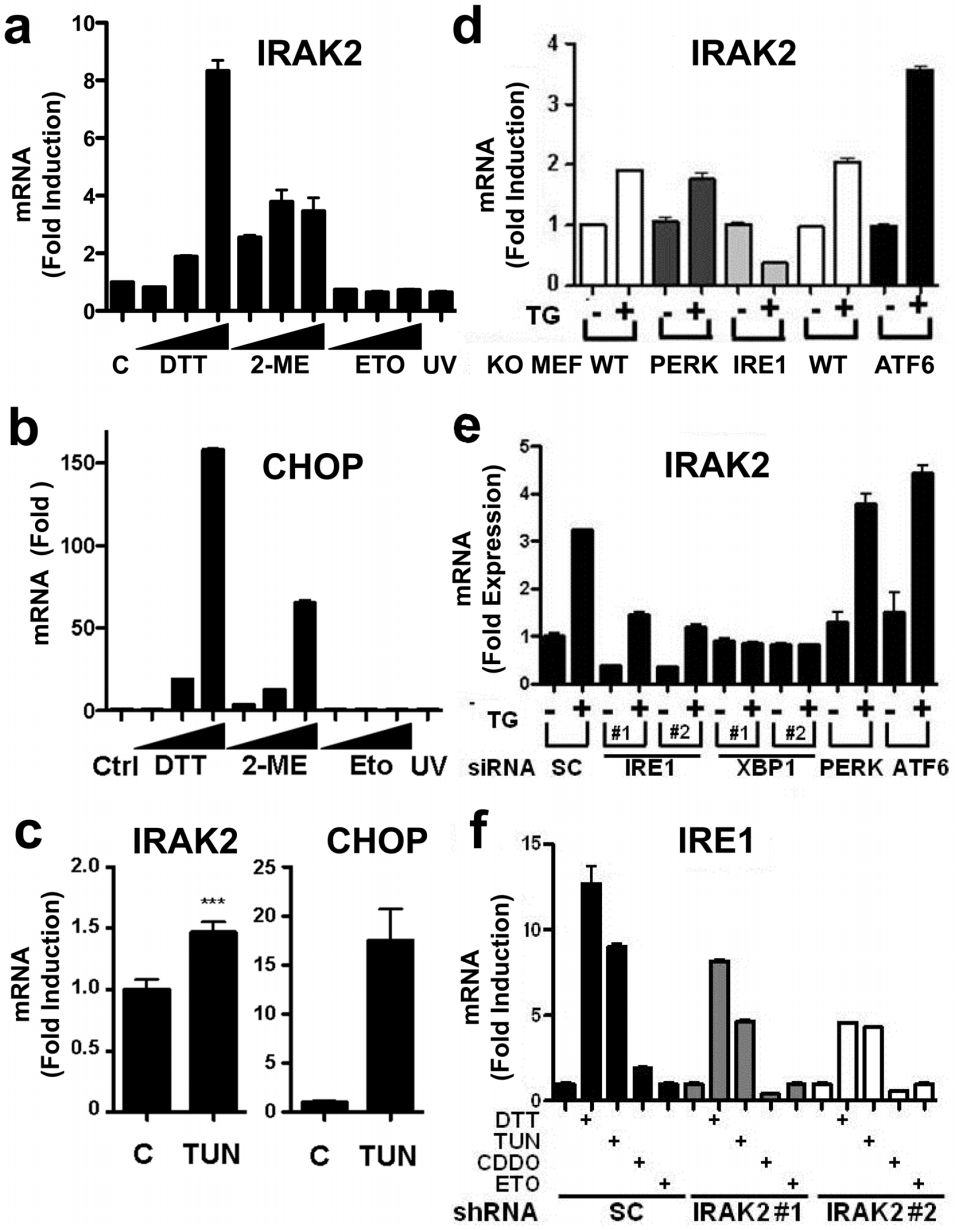
*IRAK2* gene expression is induced by the IRE1 branch of the UPR. (**a,b**) PPC1 cells were cultured with increasing concentrations of DTT (1.1–10 mM), 2-mercaptoethanol (2-ME, 5–45 mM), Etoposide (ETO: 5.5–50 µM) or exposed to 100 µJ of UV light. At 3 hrs after treatment, cells were lysed and levels of IRAK2 (a) or CHOP (b) mRNAs were measured by qRT-PCR and normalized relative to housekeeping gene, cyclophilin (mean±SD; n = 3). (**c**) Sibling wild-type mice matched for sex were given a single 1 µg/gram body weight injection of Tunicamycin (TUN). At 10 h post-injection, animals were sacrificed and their livers collected. RNA was extracted and levels of IRAK2 (left) and CHOP (right) mRNAs were measured by qRT-PCR and normalized relative to housekeeping gene, cyclophilin (mean±SD; n = 5 per group). ***Highly significant difference (by one-way ANOVA, p<0.01). (**d**) *Ire1* ^−/−^, *Perk* ^−/−^ and *Atf6* ^−/−^ mouse embryonic fibroblasts were cultured with 5 µM TG for 3 hrs. Cells were then lysed, total RNA extracted, and IRAK2 mRNA expression measured by qRT-PCR, with normalization relative to housekeeping gene, cyclophilin (mean±SD, n = 3). (**e**) PPC1 cells were transfected with 2 independent siRNAs for XBP1, CHOP, PERK and ATF6. After 2 days, cells were cultured with 5 µM TG for 3 hrs, then RNA was isolated for measuring IRAK2 mRNA levels as above (mean±SD; n = 3). Controls are available in Fig. S4 in File S1. (**f**) PPC1 cells stably infected with 2 independent IRAK2 (#1 and #2) (gray and white bars) or scrambled (SC) (black bars) shRNAs were cultured with 5 µM TG, 50 µM TUN, 0.4 µM CDDO-Im, or 100 µM of Etoposide (Eto) for 3 hrs. Cells were then lysed and total RNA was extracted. IRE1 mRNA levels were measured by qRT-PCR and normalized relative to housekeeping gene, cyclophilin (mean±SD, n = 3).

When evaluating the efficacy of siRNA and shRNA reagents on IRAK2 mRNA expression, we noticed that ER stress induces an increase in *IRAK2* gene expression (Fig. 4b, c). ER stress agents TG, CDDO-Im, DTT, and 2-mercaptoethanol (2-ME) induced concentration-dependent increase in IRAK2 mRNA levels in cultured PPC-1 cells, while DNA damaging agent Etoposide and UV irradiation had no effect (Fig. 5a,and Fig. S3b in File S1). Similarly, ER stress agents induced dose-dependent increase in UPR marker CHOP, while Etoposide and UV did not (Fig.5b). In contrast, ER stress agents did not induce expression of IRAK1 (Fig. S3b in File S1). Increases in IRAK2 mRNA following stimulation with ER stress agents were evident within 3 hrs after treatment (Fig. S3b in File S1).

To investigate whether ER stress stimulates *Irak2* gene expression *in vivo*, we induced ER stress in mice by injecting animals with Tunicamycin (TUN). Mice were then sacrificed 12 hrs later and liver tissue was harvest for total RNA extraction and analysis by qRT-PCR. Similar to cell culture experiments, mice treated with TUN showed a significant increase in IRAK2 mRNA levels compared to mice injected with the vehicle control (Fig. 5c).

### The IRE Pathway is Responsible for *IRAK2* Gene Expression during ER Stress

To identify the branch of the ER stress response responsible for inducing *Irak2* gene expression, we treated *Ire1* ^−/−^, *Perk* ^−/−^ and *Atf6* ^−/−^ mouse embryonic fibroblasts with TG, then measured the IRAK2 mRNA levels by qRT-PCR. Interestingly, the ability of ER stress to stimulate *Irak2* gene expression was completely ablated in *Ire1* ^−/−^cells, whereas IRAK2 mRNA induction was normal in *Perk* ^−/−^ and *Atf6* ^−/−^ cells (Fig. 5d). In fact, TG stimulated decreases rather than increases in IRAK2 mRNA in *Ire1* ^−/−^ cells.

To independently corroborate these findings, we employed siRNA transfection in human cells to silence proximal components of the UPR machinery, then measured IRAK2 mRNA expression following treatment with TG. Knocking down either IRE1 or XBP1 expression using two independent siRNAs for each target markedly suppressed TG-induced IRAK2 expression, whereas siRNAs targeting PERK or ATF6 were inactive (Fig. 5e). Analysis by qRT-PCR verified effective knockdown of the intended targets (Fig. S4b in File S1). We conclude therefore that the IRE1 pathway is responsible for stimulation of *Irak2* gene expression during ER stress.

Interestingly, we also noticed that the ability of ER stress to induce increases in IRE1 mRNA expression was blunted in IRAK2 knockdown cells. For example, in PPC1 cells stably expressing IRAK2 targeting shRNAs, the induction of IRE1 mRNA expression by TUN, DTT, and CDDO-Im was reduced compared to scrambled shRNA control cells (Fig. 5f). Thus, IRE1 is required for induction of IRAK2 mRNA expression and conversely IRAK2 is required for induction of IRE1 mRNA expression during ER stress.

### Reduced UPR Signaling in *IRAK2* Gene Knockout Mice

**Figure 6:**
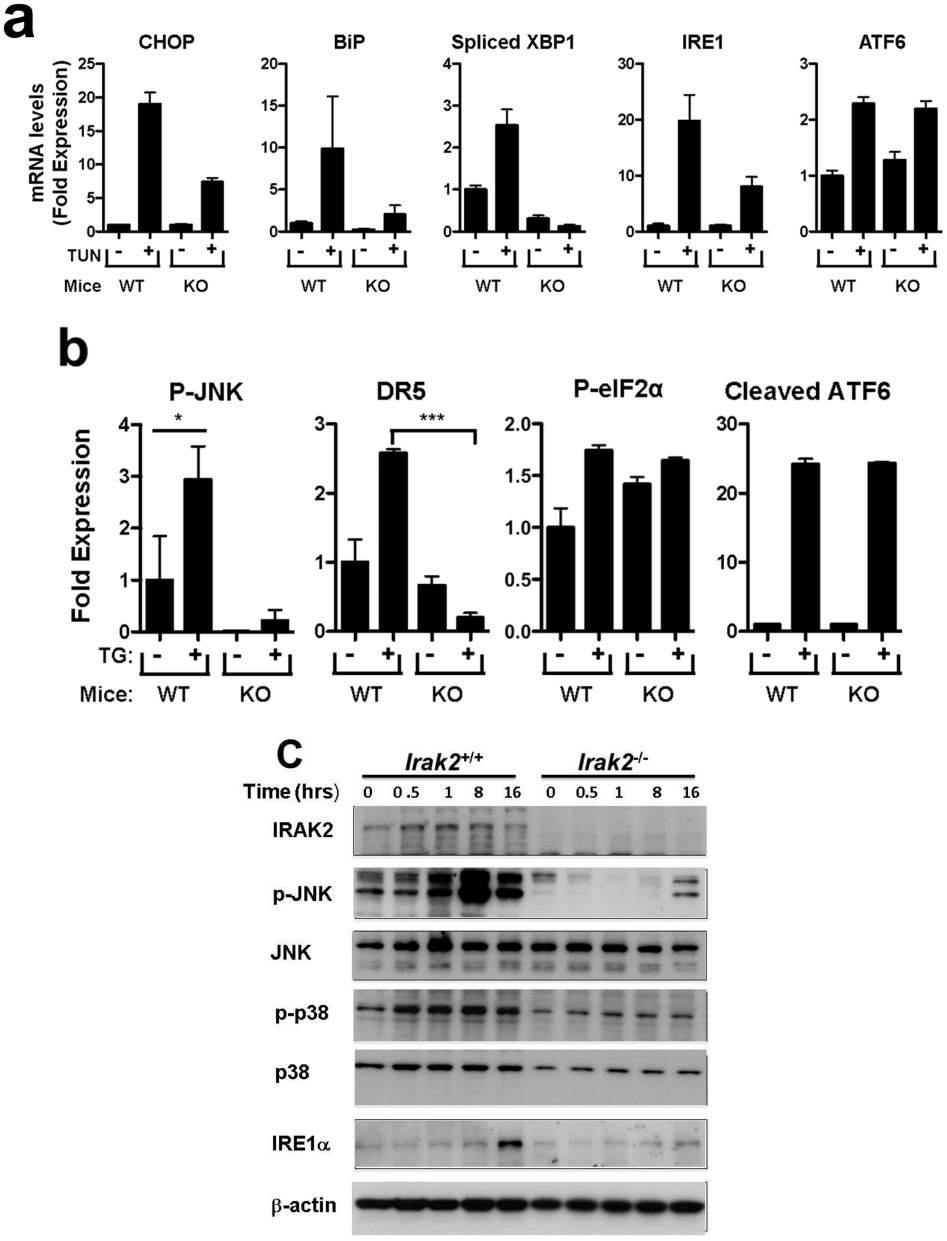
IRAK2 knockout mice exhibit reduced UPR responses. (**a**)*Irak2^+/+^*and *Irak2* ^−/−^ mice were administered 1 µg/gram body weight of Tunicamycin. At 10 hrs post injection, animals were sacrificed and their livers collected. RNA was extracted and mRNA levels of CHOP, BiP, spliced XBP1 (relative to total XBP1), IRE1, and ATF6 were analyzed by qRT-PCR, normalizing results relative to housekeeping gene, cyclophilin (mean±SD; n = 3 mice per group). (**b**) Proteins extracted from livers of *Irak2^+/+^* and *Irak2* ^−/−^ mice (50 or 100 µg) were analyzed by immunoblotting using antibodies specific for phospho-c-JNK1, c-JNK1, phospho-eIF2α, eIF2α, DR5, and ATF6. Actin served as a loading control. Immunoblot data were quantified, normalized for Actin, and data compared relative to untreated wild-type mice. For P-JNK1 and P-eIF2α, results were additionally normalized relative to JNK1 and eIF2α protein levels, respectively. For ATF6, relative levels of the cleaved protein are reported. *: p<0.05. (**c**) *Irak2* ^−/−^ and *Irak2* ^+/+^ MEFs were cultured for various times with 5 µM TG. Cell lysates were normalized for total protein content and analyzed by SDS-PAGE/immunoblotting using antibodies specific for murine IRAK2, JNK, p38MAPK, IRE1, β-Actin, and antibodies for phospho-JNK (p-JNK) and phospho-p38MAPK (p-p38MAPK). Note that while p38MAPK levels are lower in IRAK2-deficient MEFs, the ratio of p-p38MAPK to p38MAPK protein nevertheless reveals a relative diminution in p38MAPK phosphorylation in *Irak2* ^−/−^ relative to *Irak2* ^+/+^ cells.

To investigate the role of IRAK2 in ER stress signaling *in vivo*, we treated *Irak2* ^+/+^ and *Irak2* ^−/−^ littermate mice with TUN, then measured UPR signaling events in liver at the mRNA level by qRT-PCR and at the protein level by immunoblotting. *Irak2* knockout mice showed reduced induction of CHOP, BiP, and IRE1 mRNA expression and reduced XBP1 mRNA splicing compared to wild-type mice following treatment with TUN (Fig. 6a). In contrast, ATF6 mRNA levels were not different. *Irak2* knockout mice also showed reduced phosphorylation of c-JUN protein and decreased expression of the DR5 protein, compared to wild-type animals, whereas phosphorylation of eIF2α protein (PERK pathway target) and cleavage of ATF6 were not different (Fig. 6b). Overall, these results establish IRAK2 as a mediator of ER stress signaling *in vivo*, and suggest a specific role for IRAK2 in IRE1 pathway signaling.

We supplemented the studies of *Irak2* ^−/−^ mice with cell culture experiments using murine fibroblasts from *Irak*2^−/−^ and *Irak2* ^+/+^ embryos (MEF cells). Stimulation of stress kinase phosphorylation (JNK, p38) by ER stress induces was markedly reduced in *Irak*2^−/−^ cells compared to *Irak2* ^+/+^ cells (Figure 6c). *Irak2* ^−/−^ cells also had lower levels of phospho-JNK and phospho-p38 MAPK prior to stimulation, which we presume is a reflection of background ER stress caused by culturing cells in atmospheric oxygen. Of note, ER stress inducer Thapsigargin induced an increase in IRE1α protein levels in *Irak2* ^+/+^ but not *Irak2* ^−/−^ MEFs, thus confirming a role for IRAK2 in modulating expression of this UPR signaling protein (Figure 6c).

We also confirmed portions of these results *in vitro* using cultured mouse embryo fibroblasts (MEFs). TG-induced ER stress signaling was blunted in *Irak2* ^−/−^ MEFs compared to *Irak2* ^+/+^ cells, as evidenced by reductions in phosphorylation of JNK and p38MAPK and by reductions in IRE1 protein expression (Fig. 6c). Thus, IRAK2 is required for some components of the UPR, particularly the IRE1 pathway.

## Discussion

We mined the kinome by RNAi screening for novel contributors to ER stress-induced cell death, identifying IRAK2 as an amplifier of IRE1 signaling. IRE1 is known to possess a dual function in response to ER stress. First, by splicing XBP1 mRNA it enables formation of active XBP1 and the transcriptional regulation of UPR genes including CHOP, a pro-apoptotic transcription factor that regulates the expression of several death signaling molecules, including the death receptor DR5 and Bim [17], [18]. Second, by recruiting ASK1 via the adaptor TRAF2, it enables the phosphorylation of the stress kinase JNK, which in turn: (i) activates pro-apoptotic Bim and Bad proteins while inhibiting anti-apoptotic protein Bcl-2; and also (ii) translocates to the nucleus to activate the transcription factor complex AP-1, which contributes to induction of pro-apoptotic genes such as TNF-α, Fas-L and Bak [6], [19].

It is likely that our kinome screen for ER stress modulators failed to identify additional factors that may play an active role in ER stress. All screening methods introduce biases resulting from the choice of cell line, ER stress-inducing agent, time of incubation, cutoff for selecting hits (in our case 50% rescue), variations in the efficiency of siRNA-mediated gene knockdown, and other factors. Candidates such as ASK1 may have been discarded for not reaching the threshold despite the fact that they are *bona fide* regulators of ER stress. Also, given that many ER stress pathways are redundant, the absence of one kinase may not have been sufficient to meet or exceed the threshold set for rescuing from ER stress-induced cell death. Importantly, in our secondary screens, 4 candidates were discarded for failure to suppress induction of CHOP activity. Such candidates may otherwise influence ER stress without regulating CHOP activity.

We have shown that upon ER stress, IRAK2 is required for upregulation of IRE1 mRNA expression, thus amplifying IRE1 activities including XBP1 splicing and stress kinase activation. Conversely, we have shown that ER stress via IRE1 robustly induces the expression of IRAK2. Therefore, IRAK2 is part of an amplification loop whereby ER stress-induced activation of IRE1 causes IRAK2 mRNA expression, which in turn induces more IRE1 expression, thereby amplifying IRE1 activity and sustaining the ER stress response (depicted in Fig. S5 in File S1).

Unlike family members IRAK1 and IRAK4, it is controversial whether IRAK2 has intrinsic kinase activity and some have described IRAK2 as a pseudokinase [20]. Kinase domains require 3 functional motifs for ATP binding and catalytic activity. Inspection of the amino-acid sequences of human and mouse IRAK2 reveals apparent absence of two of the three motifs required for ATP binding and catalytic activity, including the conserved aspartic acid residue necessary for catalytic activity [8]. Nevertheless, mutagenesis studies of the putative ATP-binding pocket of mouse IRAK2 protein (affecting the one well-conserved kinase motif) have suggested that ATP binding is required for IRAK2 to become phosphorylated in cells and that it is also necessary for IRAK2-mediated signal transduction [9], [21]. However, even the ATP site mutant of murine IRAK2 retains considerable signal transduction function in cells [9], [21], strongly arguing that it possesses kinase-independent activity. Interestingly, of the two fly IRAK homologs, only the sequence of Pelle contains the necessary features for kinase activity. In contrast, Tube entirely lacks the kinase domain [22] and yet we found that Tube(not Pelle) mediates the ER stress response in fly cells.

We therefore speculate that IRAK2 may regulate IRE1 expression by acting as an adaptor protein. In the TLR signaling pathway, IRAK2 is known to act as an adaptor between the TLR-MyD88-IRAK4 complex and TRAF6, enabling the downstream activation of NF-κB [23]. With regards to ER stress, IRAK2 potentially could operate at several levels to impact IRE1 signaling. First, because we found that IRAK2 is required for ER stress-induced increases in IRE1 mRNA and protein levels, IRAK2 might collaborate with signaling enzymes or transcription factors that control *IRE1*gene expression. Second, it is possible that IRAK2 acts as an adaptor for the IRE1 protein by directly or indirectly interacting with unknown proteins and recruiting them to the IRE1 protein complex to enhance XBP1 splicing and stress kinase activation.

Our findings highlight the role of IRAK2 in ER stress in addition to its role in innate immunity. Several reports have previously hinted at a link between innate immunity and ER stress. For instance, the activation of Toll-like receptors (TLRs) has been shown to induce the ER unfolded protein response [24]. In addition, virus infection of monocytic cells can activate XBP1 splicing and Grp78 (Bip) expression [25]. In *C. elegans,* innate immunity pathways were shown to be physiologically relevant inducers of ER stress, where activation of the response to infection with certain bacteria induces XBP-1-dependent UPR responses [26]. The link between immune responses and ER stress has also been established in pathological paradigms. Hereditary polymorphisms in the gene encoding XBP1 have been associated with inflammatory bowel disease (IBD), a condition that is believed to arise from dysregulation in host immune responses to enteric microflora [27]. Therefore, IRAK2 could serve as a linking protein that enables cross-talk between ER stress and innate immunity signaling processes.

Because ER stress has been implicated in the pathogenesis of multiple diseases, including neurodegeneration, diabetes, IBD, and cancer, IRAK2 represents a potential new drug target. However, lacking the features required for ATP binding and kinase activity, the “druggability” of human IRAK2 is questionable. Instead of traditional strategies that target the ATP binding pocket of kinases, approaches to therapeutic targeting of IRAK2 may require either disrupting protein interactions in which IRAK2 participates or targeting IRAK2 mRNA expression with antisense DNA or siRNA drugs. Further elaboration of the roles of IRAK2 in animal models of disease where ER stress has been implicated are needed to reveal the efficacy and safety of targeting IRAK2 with therapeutic intent.

## Methods

Additional experimental procedures are provided as supplementary information.

### Reagents and Cell Lines

CDDO-Imidazole (2-cyano-3,12 dioxooleana-1,9 dien-28-oyl imidazole) was provided by the National Cancer Institute, dissolved in DMSO, and aliquots stored at −80°C. Salubrinal (ID-5747990, 3-phenyl-N-(2, 2, 2-trichloro-1-{[(8-quinolinylamino) carbonothioyl] amino} ethyl) acrylamide) was obtained from ChemBridge (San Diego). Staurosporine, Cycloheximide, Tumor Necrosis Factor-α (TNF), Tunicamycin, DTT, Etoposide, Brefeldin A, PMA and 2-mercaptoethanol were purchased from Sigma. G0 6983 was purchased from Sagria. Thapsigargin was purchased from Axxora (San Diego). CHOP-Luc plasmid construct was kindly provided by Prof. Pierre Fafournoux, France [28]. The pFA-CHOP and pFA-c-JUN chimeric plasmids were obtained from Agilent (PathDetect).

Human PPC1 prostate cancer cells [13] were cultured in RPMI with 10% fetal bovine serum (FBS). HeLa, A549, ALVA-31 and HEK293T cell lines [13], [29], as well as wild-type and *Ire1* ^−/−^, *Perk* ^−/−^, *Atf6* ^−/−^, and *Irak2* ^−/−^ mouse embryonic fibroblasts[6], [30]–[33] were cultured in DMEM with 10% FBS. *Drosophila* S2cells were maintained in Schneider’s *Drosophila* medium (Invitrogen) supplemented with 10% FBS (nutrient full medium).

### RNAi Library Screen

The kinase-focused library of siRNAs was derived from the Ambion druggable V3 library and covers all known kinases (n = 714) with 4 different siRNAs against each target. The siRNAs were spotted in 384 well plates and reverse-transfected into PPC1 cells at 1000 cells/well for 48 hrs. Subsequently, cells were treated for 24 hrs with 1.5 µM of the imidazole derivative of the compound CDDO, which is known to induce tumor cell apoptosis via ER stress [12]. Cell viability was assessed by measuring ATP levels using a bioluminescence assay (ATPlite [Perkin-Elmer]). Hits were selected based upon the criteria of (a) >50% protection against CDDO-Im induced cell death and (b) minimum of 2 siRNAs showing protective activity.

### Gene Knockdown by RNAi Transfection

Human cells were transfected with 10 nM siRNAs using Lipofectamine RNAiMax (Invitrogen) in OptiMEM medium (Invitrogen). At 24 hrs after transfection, the transfection medium was replaced with the fresh culture media and the cells were cultured for 48 hrs. Human siRNAs used were purchased from Ambion, USA: IRAK2 ID: S139, S140, S141 and S7495; XBP1: S14914 andS14915;IRE1: S200430 and S200431; PERK: S18102; ATF6: S223543; CHOP: 146321 and 146320.

Gene knockdown in S2 fly cells was achieved using RNA interference. Briefly, dsRNAs were generated by reverse transcription (T7 RiboMax Express RNAi systems from Promega) of PCR products. pFR-luciferase stable S2 cells were re-suspended in Schneider’s Drosophila medium (Invitrogen) supplemented with 10% dialyzed FBS (Signal) and seeded at 20,000 cells per well in a 384 well plate. Cells were then transfected with pFA-c-JUN or pFA-CHOP (0.03 µg) and dsRNA (0.08 µg) using Effectene (Qiagen), incubated for 72 hrs at 25°C and treated with Thapsigargin or DMSO for an additional 24 hrs. Subsequently, 30 µL of Bright-Glo (Promega) was added to each well and luminescence was measured using EnVision (PerkinElmer). Fly PCR primers contained a 5′ T7 RNA polymerase binding site (TAATACGACTCACTATAGG) followed by sequences specific for various targeted genes (see File S1).

### Gene Knockdown by Stable shRNA Vectors

Stable PPC1 cells harboring shRNAs targeting human IRAK2 (2 independent shRNAs) or a scrambled control were established by means of lentiviral infection using vectors obtained from Sigma-Aldrich, USA (TRCN0000197065 and TRCN0000199667, containing the sequences GTTCGCCTCCTACGTGATCAC and GAAACAGACGACGTTGACAA, respectively). The lentiviruses were generated by concomitant transfection of three independent plasmids (envelope, integration-replication, and shRNA cassette containing plasmid) into HEK293T cells as previously described [34]. Virus-containing culture supernatants were harvested 48 hrs later. PPC1 cells were infected by replacing their media with viral supernatants. The procedure was repeated 24 hrs later. After infection, stably infected clones were selected by puromycin treatment for 5 days. Individual clones were subsequently isolated and cultured individually, then tested for IRAK2 knockdown efficiency by qRT-PCR.

### Cell Viability

Cell viability was measured using the ATPlite 1-step Luminescence assay system (PerkinElmer) in which ATP serves as a surrogate for cell viability. In brief, 25 µL/well (384 wells plates) ATPlite solution was added and the plates were kept in dark for 5 minutes. Luminescence was measured using EnVision Multilabel Plate Reader (PerkinElmer). Results were confirmed by Caspase-3/7 activity (DEVDase) assay and by DAPI staining [34].

### Luciferase Assays

Mammalian cells in 12 well dishes were co-transfected using RNAiMax (Invitrogen) with 3.2 µg of CHOP-Firefly Luciferase plasmid and 0.07 µg of pRL *Renilla* Luciferase Control Reporter (Promega) for 4–6 hrs. Luciferase activity was measured using the DualGlo luciferase assay kit (Promega).

For fly cells, S2 cells (available from Invitrogen) stably containing PathDetect pFR-luciferase encoding plasmid (Agilent) were re-suspended in Schneider’s Drosophila medium (Invitrogen) supplemented with 10% dialyzed FBS (Signal) and seeded at 20,000 cells per well in 384 well plates. Cells were then transfected with PathDetect plasmids: 0.03 µg of pFA-c-JUN (encoding the transactivation domain of c-JUN fused to the Gal4 DNA-binding domain) or 0.08 µg of pFA-CHOP-Luc (encoding CHOP transactivation domain fused to Gal4 DNA-binding domain) and dsRNAs using Effectene (Qiagen), incubated for 72 hrs at 25°C and treated with Thapsigargin, Brefeldin A, CDDO-Im, phorbol-12-myristate-13-acetate (PMA), the PKC inhibitor G0 6983 (Sagria), or DMSO for an additional 24 hrs. Subsequently, 30 µL of Bright-Glo (Promega) was added to each well and luminescence was measured using EnVision plate reader (PerkinElmer).

### Caspase-3/7 Activity

Caspase-3/7 activity was measured using the Caspase-Glo 3/7 activity assay (Promega) according to the manufacturer’s instructions. Briefly, Caspase-Glo 3/7 substrate was added to 384 well plates (50 µL/well) containing 1000 cells cultured in 50 µL of complete medium. The plates were incubated for 30 minutes, placed onto a rocking platform for 2 minutes, and then read using a luminometer (Luminoskan Ascent; Therom Electron Corp.).

### Quantitative RT-PCR

Total RNAs were extracted using RNeasy columns for cultured cells (Qiagen) and Trizol for tissue samples (Invitrogen). Subsequently, 3 µg RNA per sample was used for reverse transcription using Roche-Transcriptor first strand according to the manufacturer’s instructions (Roche, USA), then 2 µL of the resulting RT product was used for Q-PCR using 7.5 µM of specific primers (see File S1) in conjunction with Roche SYBR Green I Master Mix and the LightCycler 480 instrument (Roche, USA).

### Immunoblotting

Protein concentrations were measured by Bicinchoninic acid (BCA) assay (Thermo) before addition of 4x Laemmli sample buffer. Samples were subsequently boiled for 5 minutes, run on 4–20% gradient gels (Life Technologies), transferred to nitrocellulose membranes using the Trans-Blot Turbo system (Bio-Rad) and immunoblotted using various antibodies that included Phospho-JNK, JNK, ATF6, DR5 from Cell Signaling (USA) and Phospho-eIF2α from Enzo Life Sciences (USA). Actin was used as a loading control. Antibodies were detected using secondary Horse Radish Peroxidase (HRP)-conjugated antibodies. Immunoblots were quantified using imageJ software.

### Animal Studies

*In vivo* work was pursued upon approval by the Institutional Animal Care and Use Committee (IACUC) of the Sanford-Burnham Medical Research Institute. *Irak2* ^−/−^ mice were generated as previously described [35]. Sibling mice (6- to 10-week-old), matched for sex were administered a single 1 µg/g body weight intraperitoneal (ip) injection of a 0.05 mg/ml suspension of Tunicamycin in 150 mM dextrose. At 10 hrs post injection, animals were sacrificed by Avertin overdose (0.34 ml/g IP) prior to cervical dislocation for organ collection.

## Supporting Information

File S1 (PDF)
