# Supplementary material for: Interleukin-1 Receptor-Associated Kinase-2 (IRAK2) Is a Critical Mediator of Endoplasmic Reticulum (ER) Stress Signaling
Source: PLoS One. 2013 May 28;8(5):e64256. doi: 10.1371/journal.pone.0064256 (PMC3665826; doi:10.1371/journal.pone.0064256)
Supplement: File S1 — (PDF) [file pone.0064256.s001.pdf]

SUPPORTING INFORMATION

I. Experimental Methods:

RNAi reagents for gene silencing in fly S2 cells.

The following primers were used for PCR amplification of RNAs for gene silencing in fly S2 cells.

Tub_dsRNA_Fw: TAATACGACTCACTATAGGCCCTGCCCGGCCTGT

Tub_dsRNA_Rev: TAATACGACTCACTATAGGTGATTCGCGGAATGTTAGG

Tub_dsRNA_2_Fw: TAATACGACTCACTATAGGAATGACATTTACCGGCTTGC

Tub_dsRNA_2_Rev: TAATACGACTCACTATAGGCTCGTTGTCCACTTCCATT

Pll_dsRNA_Fw: TAATACGACTCACTATAGGGGGCGTCAGCAACTCAA

Pll_dsRNA_Rev: TAATACGACTCACTATAGGCCGAGGCTGATGCTAAAC

Pll_dsRNA_2_Fw: AATACGACTCACTATAGGCCGTGTGATTGTACTGACCG

Pll_dsRNA_2_Rev: TAATACGACTCACTATAGGACAGGTTTTCATCGAATCGC

Ire1_dsRNA_Fw: TAATACGACTCACTATAGGAACACGCCTCGTATTTCCAC

Ire1_dsRNA_Rev: TAATACGACTCACTATAGGAGGCAATCATACCCAGAACG

qRT-PCR primers.

The thermal amplification profile was as follows: Activation: 95°C for 5 minutes for 1 cycle | Amplification: 95°C for 10 sec, 60°C for 10 sec and 72°C for 10 sec for 45 cycles | Final amplification: 95°C for 5 sec, 60°C for 1 minute and 1 cycle of cooling at 40°C for 30 sec. Q-PCR results were considered valid when efficiency E>95% and standard curve correlation R^2>99%

were achieved. The quality of the resulting PCR products was assessed by thermal melting point determination.

The sequences of DNA primers used for qRT-PCR analysis of transcripts were as follows:

Human CHOP Fwd: CAGAGCTGGAACCTGAGGAG Rev: TGGATCAGTCTGGAAAAGCA

Human XBP1 total Fwd: TGACGAGGTTCCAGAGGTG Rev: TGCAGAGGTGCACATAGTCTG

Human XBP1 unspliced Fwd: CCGCAGCACTCAGACTACG Rev: ATGTTCTGGAGGGGTGACAA

Human IRAK2 Fwd: CCTCCTCTGAGGCCTGTGT Rev: TGATCTCAATTTGCCACGAA

Human IRAK 1 Fwd: GAGACCTTGGCTGGTCAGAG Rev: AGTGTGCTCTGGGTGCTTCT

Human BIP Fwd: CAACCAACTGTTACAATCAAGGTC Rev: CAAAGGTGACTTCAATCTGTGG

Human IRE1 Fwd: CCATCGAGCTGTGTGCAG Rev: TGTTGAGGGAGTGGAGGTG

Human PERK Fwd: CAGTGGGATTTGGATGTGG Rev: GGAATGATCATCTTATTCCCAAA

Human ATF6 Fwd: TTGGCATTTATAATACTGAACTATGGA Rev: TTTGATTTGCAGGGCTCAC

Human NQO1 Fwd: CAGCTCACCGAGAGCCTAGT Rev: GAGTGAGCCAGTACGATCAGTG

Human JUN Fwd: CCAAAGGATAGTGCGATGTTT Rev: CTGTCCCTCTCCACTGCAAC

Human ATG12 Fwd: TCTTCCGCTGCAGTTTCC Rev: GTCTCCCACAGCCTTTAGCA

Mouse CHOP Fwd: GCGACAGAGCCAGAATAACA Rev: GATGCACTTCCTTCTGGAACA

Mouse IRAK2 Fwd: AGCACAGCCATCCACCAG Rev: TGATCTCAATTTTCCATGAAGTCT

Mouse XBP1 total Fwd: AGCAAGTGGTGGATTTGGAA Rev: CCGTGAGTTTTCTCCCGTAA

Mouse XBP1 unspliced Fwd: TGACGAGGTTCCAGAGGTG Rev: TGCAGAGGTGCACATAGTCTG

Mouse BIP Fwd: CAAGTTCTTGCCATTCAAGGT Rev: CTTCTGGGGCAAATGTCTTG

Mouse IRE1 Fwd: TGAAACACCCCTTCTTCTGG Rev: CCTCCTTTTCTATTCGGTCACTT

Mouse PERK Fwd: CCTTGGTTTCATCTAGCCTCA Rev: ATCCAGGGAGGGGATGAT

Mouse ATF6 Fwd: GGACGAGGTGGTGTCAGAG Rev: GACAGCTCTTCGCTTTGGAC

For qRT-PCR analysis of fly S2 cells, the following primers were employed:

Tub _1_Fw: AGCAGAGCTCAAACAATGAC

Tub _1_Rev: CAAATGAATACATCTGGGTTG

Tub _2_Fw: ACGATGATAACGATGGTGAG

Tub _2_Rev: TGCAGCTCACTCAAGTCTG

Pll _1_Fw: CATGAATCAGTCGCAACC

Pll _1_Rev: GTCGGGATAGAGTTTTACGG

Pll _2_Fw: ACTACGTTAAGCAGCAGTGG

Pll _2_Rev: CCATCTAGTCGGTAACAAACG

XBP1_Full _Fw: TCTAACCTGGGAGGAGAAAG

XBP1_Full _Rev: GTCCAGCTTGTGGTTCTTG

XBP1_S _Fw: CAGCATCCAAAGCTGACCCTCTG

XBP1_S _Rev: ATATCTGCGAGCAGACTTTC

IRE1 _Fw: TCTCCCACGAATACAATCTG

IRE1 _Rev: GAATTGCAACTTCTCCACAC

rp49 _Fw: ATCGTGAAGAAGCGCACCAAG

rp49 _Rev: ACCAGGAACTTCTTGAATCCG

Cell Lysis

Cells were resuspended in a lysis buffer containing 20 mM HEPES (pH 7.3), 150 mM NaCl, 0.5% NP-40, 1 mM EDTA and Complete EDTA-free protease inhibitor cocktail (Roche). Lysates kept on

ice for 20 minutes were then centrifuged at 12,000g for 10 minutes to eliminate debris. Animal tissue protein lysates were prepared in a hypotonic lysis buffer containing 10 mM NaCl, 10 mM Tris-HCl pH 7.2, 1 mM EDTA, 1% Triton and Complete EDTA-free protease inhibitor cocktail (Roche). After incubation on ice for 20 min, samples were centrifuged 3 times at 12,000g for 10 minutes.

II. Supporting Figure Legends:

Figure S1: Overview of kinome screening campaign.

(A) The flow chart depicts the process used to prosecute hits from siRNA kinome library screening. See text for details. **(B)** Alva-31 and A549 cells were transfected with IRAK2, scrambled (Sc) or Caspase-8 (casp 8) siRNAs. After 2 days, cells were treated with CDDO-Im for 32 h (Alva-31) or 12 h (A549). Cell viability was measured by ATPLite assay. Non-treated controls (DMSO) were normalized to 100%. Data are mean±SD, n = 3.

Figure S2: Additional studies of effects of IRAK2 on UPR signaling.

(A) HeLa cells were transiently transfected with 2 independent IRAK2 siRNAs or scrambled controls (SC). After 2 days, cells were cultured with 5 μM TG or 100 μM Etoposide (Eto) for 3 hrs then total RNA was extracted. Levels of endogenous CHOP and IRAK2 mRNAs were measured by qRT-PCR and normalized relative to housekeeping gene, cyclophilin (mean±SD, n = 3). **(B,C)** PPC-1 cells stably expressing scrambled or IRAK2 shRNAs were cultured with 5 μM Thapsigargin (TG) for 3 hrs or 100 ng/mL TNF-α for 0-60 min. Lysates were prepared, normalized for total protein content, and analyzed by immunoblotting using antibodies specific for phosphor-JNK1, total JNK1, cleaved ATF6, or actin. Data representative of 3 or more experiments. **(D)** qRT-PCR for CHOP

mRNA was performed using samples from Fig. 4a and displayed as ratios relative to housekeeping gene, cyclophilin. Data are mean±SD, n = 3.

Figure S3: IRAK2 mRNA expression is selectively induced by ER stress.

(A) The levels of NQ01, JUN and ATF6 mRNAs were analyzed by qRT-PCR using samples from Fig. 4c and normalized relative to housekeeping gene, cyclophilin (mean±SD, n = 3). **(B)** PPC-1 cells were cultured with 5 μM TG, 5 μM CDDO-Im, or DMSO for various times, then total RNA was extracted and levels of the indicated mRNAs were analyzed by qRT-PCR with normalization relative to housekeeping gene, cyclophilin (mean±SD, n = 3).

Figure S4: Confirmation of target knockdown by RNA silencing.

(A) Lysates were prepared from *Ire1*$^{-/-}$, *Perk*$^{-/-}$ *and Atf6*$^{-/-}$ mouse embryonic fibroblasts, normalized for total protein content, and analyzed by immunoblotting using the indicated antibodies. **(B)** The levels of mRNAs for ASK1, CHOP, and XBP1 were measured in qRT-PCR in PPC-1 transfected with various siRNAs as indicated, using 2 independent siRNAs for each target. Data are mean±SD, n = 3.

Figure S5: Proposed mechanism of IRAK2 regulation of ER stress signaling.

IRE1 activation by ER stress stimulates IRAK2 gene expression via a XBP1-dependent mechanism. Reciprocally, IRAK2 promotes ER stress-induced expression of the IRE1 gene. The IRE1 pathway stimulates stress kinase activation, which is known to promote apoptosis.

a

Kinase siRNA Library
707 kinases (4x), 2900 siRNAs total

Primary screen: 25 Hits
(2 to 4 siRNAs protected cell death for each hits)
ATPlite assay-24h treatment with CDDO-Im (1.5 µM)

Secondary screen: 7 Hits
ATPlite assay-selected 7 hits which are confident

Target validation: 6 Hits
Knockdown verification by QRT-PCR**:** 6 targets validated

Pathway selectivity: 6 Hits
Staurosporin, TNF, Thapsigargin

Inhibition of CHOP activity: 2 Hits
Thapsigargin, Tunicamycin, CDDO-Im

Hit confirmation in multiple cell lines: 1 Hit
Alva-31, A549, U251

b

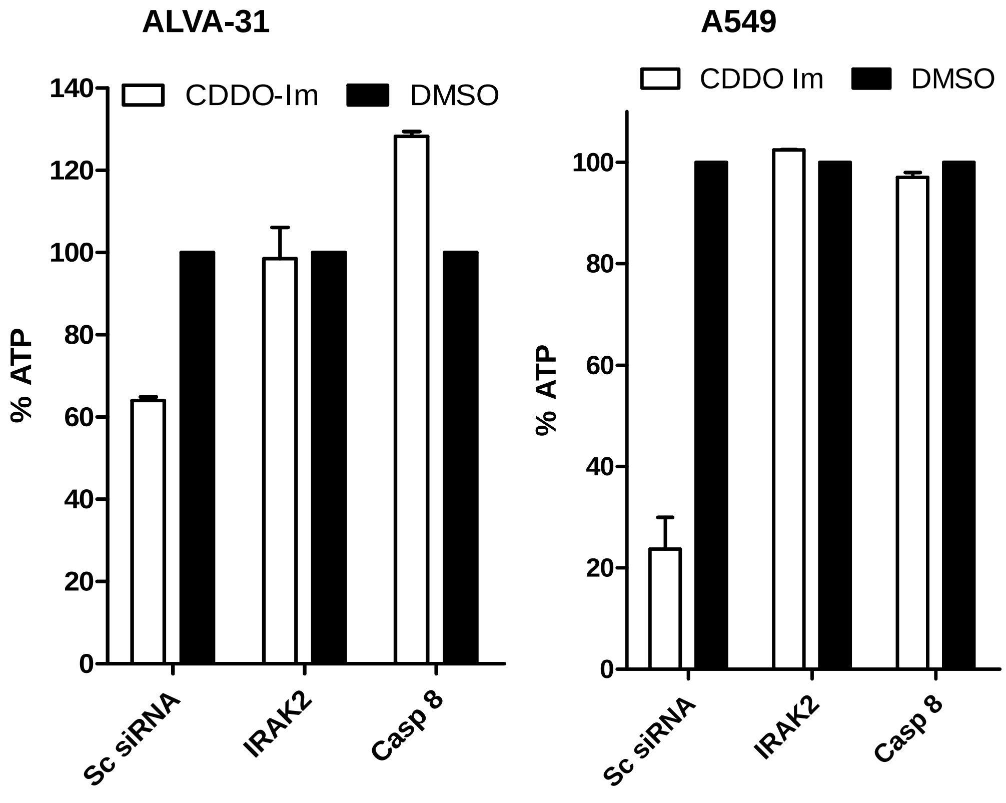

Figure S1

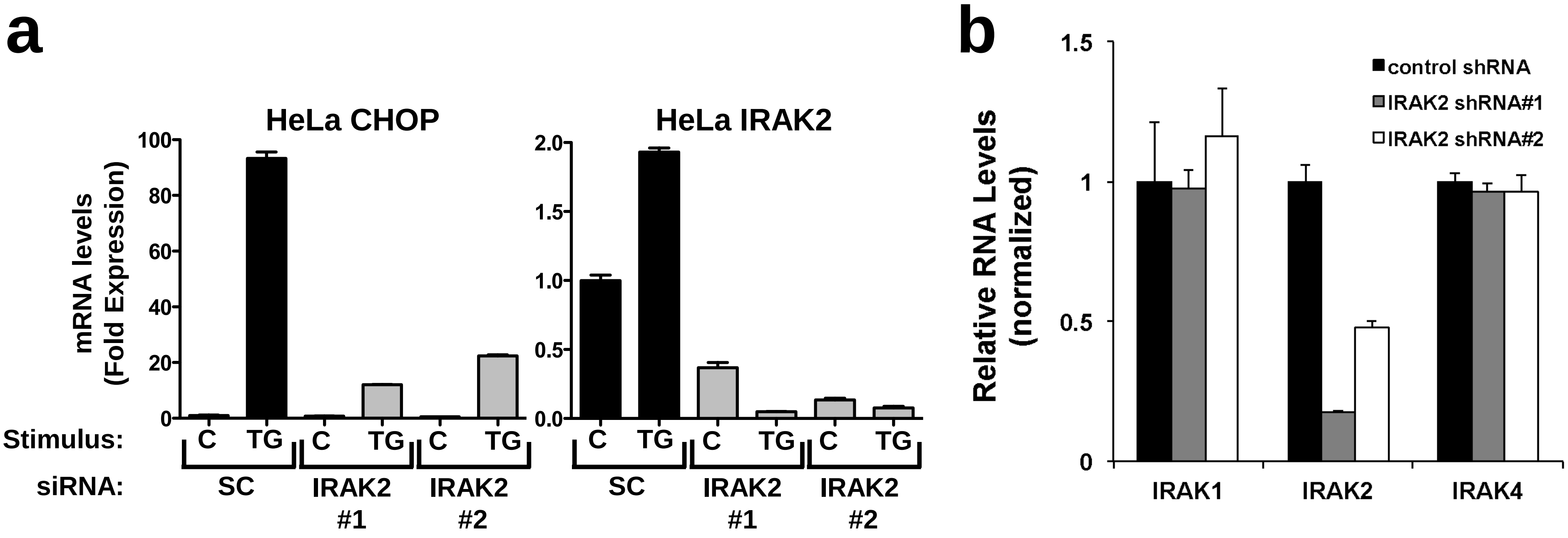
a
HeLa CHOP
HeLa IRAK2
mRNA levels (Fold Expression)
100
80
60
40
20
0
2.0
1.5
1.0
0.5
0.0
Stimulus:
C
TG
siRNA:
SC
IRAK2 #1
IRAK2 #2
b
1.5
1
0.5
0
Relative RNA Levels (normalized)
control shRNA
IRAK2 shRNA#1
IRAK2 shRNA#2
IRAK1
IRAK2
IRAK4

Figure S2

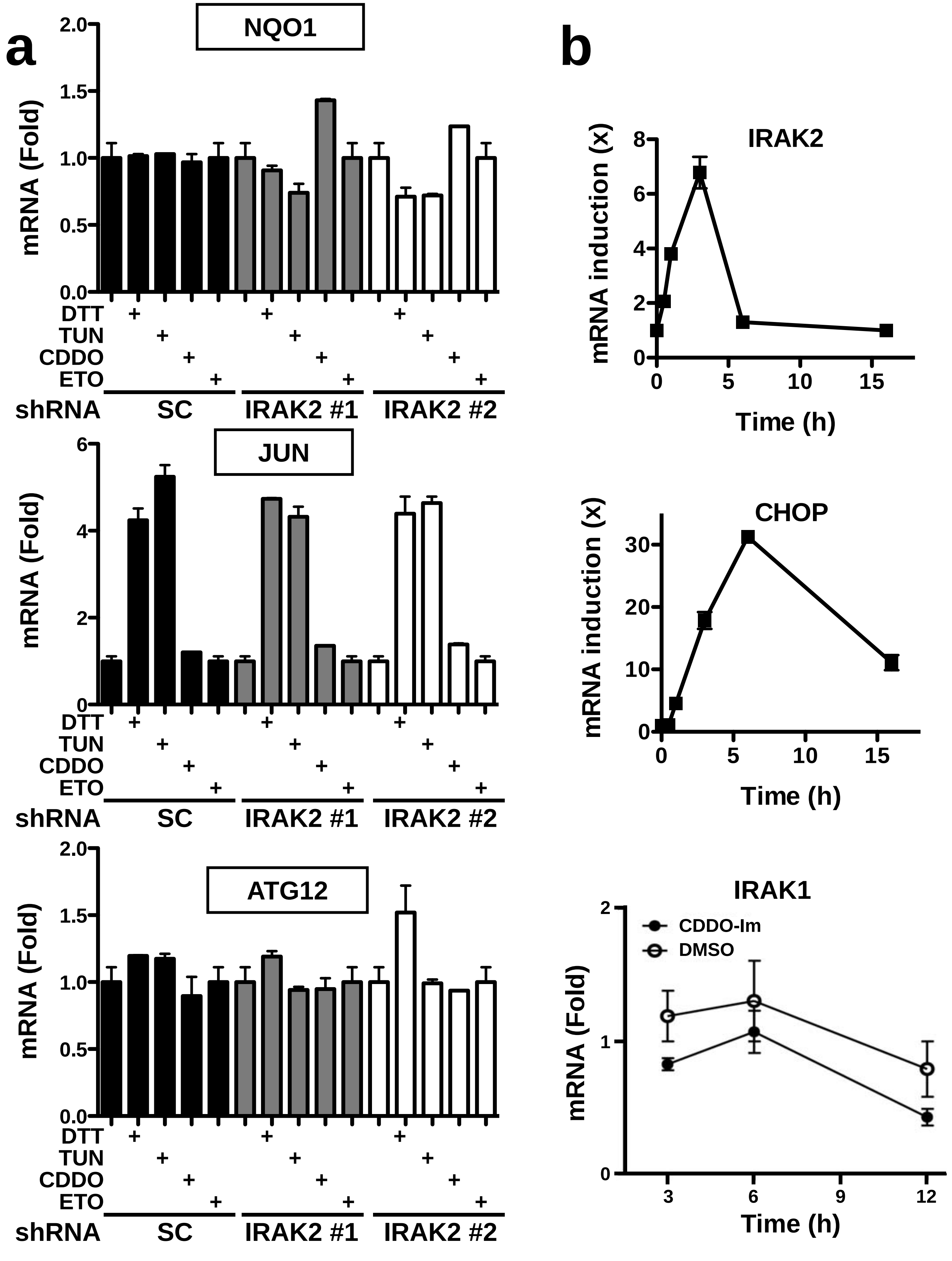
a
NQO1
mRNA (Fold)
2.0
1.5
1.0
0.5
0.0
DTT
TUN
CDDO
ETO
shRNA
SC
IRAK2 #1
IRAK2 #2
JUN
6
4
2
0
ATG12
b
IRAK2
mRNA induction (x)
8
6
4
2
0
5
10
15
Time (h)
CHOP
30
20
10
IRAK1
CDDO-Im
DMSO
3
6
9
12

Figure S3

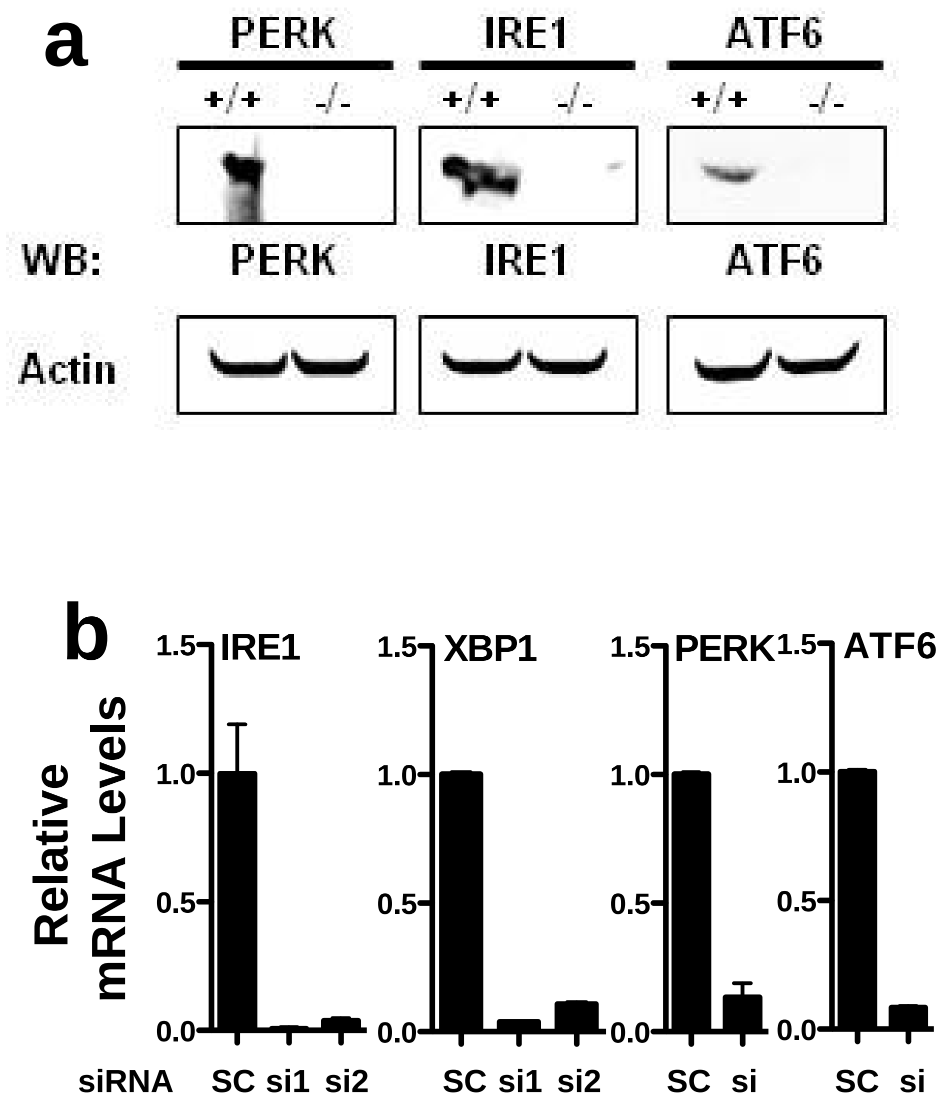
a
PERK
IRE1
ATF6
+/+
-/-
WB:
Actin
b
Relative mRNA Levels
1.5
1.0
0.5
0.0
XBP1
siRNA
SC
si1
si2
si

Figure S4

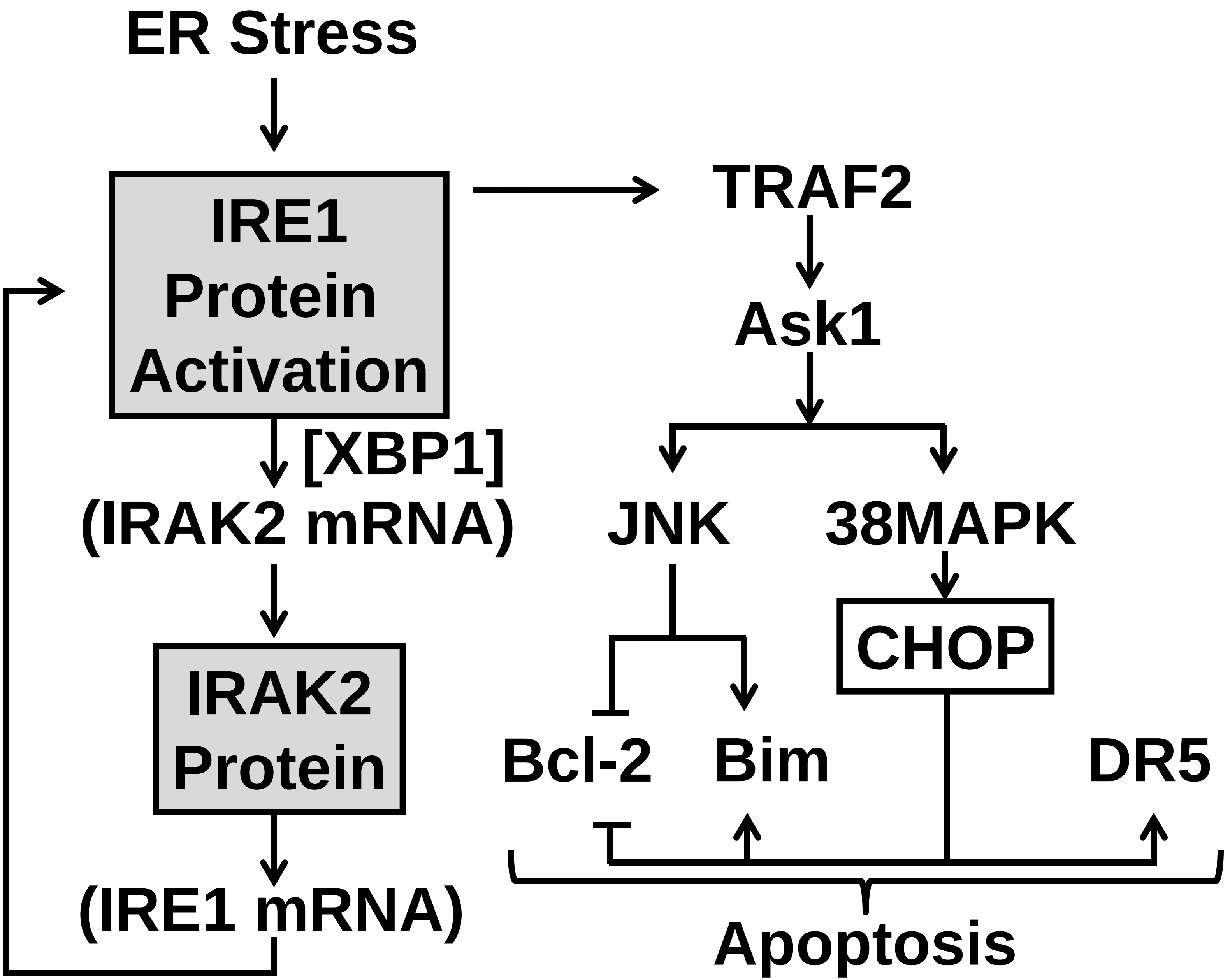
ER Stress
IRE1
Protein
Activation
TRAF2
Ask1
[XBP1]
(IRAK2 mRNA)
JNK
38MAPK
CHOP
IRAK2
Protein
Bcl-2
Bim
DR5
(IRE1 mRNA)
Apoptosis

Figure S5
